# Efficient computation of minimal perturbation sets in gene regulatory networks

**DOI:** 10.3389/fphys.2013.00361

**Published:** 2013-12-17

**Authors:** Abhishek Garg, Kartik Mohanram, Alessandro Di Cara, Gwendoline Degueurce, Mark Ibberson, Julien Dorier, Ioannis Xenarios

**Affiliations:** ^1^Vital-IT Systems Biology Division, SIB Swiss Institute of BioinformaticsLausanne, Switzerland; ^2^Electrical and Computer Engineering, University of PittsburghPittsburgh, PA, USA; ^3^Quartz BioPlan-Les-Ouates, Switzerland; ^4^Center for Integrative Genomics, University of LausanneLausanne, Switzerland; ^5^Swiss-Prot group, SIB Swiss Institute of BioinformaticsLausanne, Switzerland

**Keywords:** boolean modeling, GRN, MIS, miRNA, algorithms, qualitative modeling, T-Helper, cancer pathways

## Abstract

In the last few decades, technological and experimental advancements have enabled a more precise understanding of the mode of action of drugs with respect to human cell signaling pathways and have positively influenced the design of new drug compounds. However, as the design of compounds has become increasingly target-specific, the overall effects of a drug on adjacent cellular signaling pathways remain difficult to predict because of the complexity of the interactions involved. Off-target effects of drugs are known to influence their efficacy and safety. Similarly, drugs which are more target-specific also suffer from lack of efficacy because their scope might be too limited in the context of cellular signaling. Even in situations where the signaling pathways targeted by a drug are known, the presence of point mutations in some of the components of the pathways can render a therapy ineffective in a considerable target subpopulation. Some of these issues can be addressed by predicting *Minimal Intervention Sets* (MIS) of elements of the signaling pathways that when perturbed give rise to a pre-defined cellular phenotype. These minimal gene perturbation sets can then be further used to screen a library of drug compounds in order to discover effective drug therapies. This manuscript describes algorithms that can be used to discover MIS in a gene regulatory network that can lead to a defined cellular phenotype. Algorithms are implemented in our Boolean modeling toolbox, *GenYsis*. The software binaries of *GenYsis* are available for download from http://www.vital-it.ch/software/genYsis/.

## 1. Introduction

Advancements in high-throughput technologies have enabled biologists to measure the expression (or activity) of many genes simultaneously. By measuring and comparing the expression of genes in a normal vs. diseased cell phenotypes, biologists have been able to identify various key genes involved in disease pathways. However, predicting a set of genes in a disease is often not sufficient as it may not be possible to directly manipulate the expression of those genes using available drug compounds. This necessitates studying the interactions of genes that are known drug targets with respect to the genes (or proteins) that are implicated in high-throughput analysis of specific disease phenotypes. This observation has shifted the focus of computational and experimental tools from studying individual genes to understanding the underlying gene regulatory networks (*GRNs*) of biological processes. By modeling GRNs, one would like to gain a deeper understanding of how different cellular phenotypes arise from the same set of underlying genes and how a biological system can be forced to differentiate into a specific phenotype by manipulating the expression of a small set of genes/proteins in the underlying GRN.

Even with an improved understanding of the working of cellular signaling components and technological advancements in designing new drug compounds, it is often not possible to design drug compounds that specifically target only the desired genes/proteins with desired effectiveness. Some of these issues can also be addressed by predicting which elements of the GRNs have to be targeted in order to attain a pre-defined phenotype. The latter problem is formally referred to as *Minimal Intervention Sets* (MIS) problem in the literature (Karlebach and Shamir, 2010; Samaga et al., 2010). In an MIS problem, one would like to enumerate a list of possible sets of genes/proteins from a given GRN, which when perturbed, can give rise to the desired cellular phenotypes.

**Figure 1:**
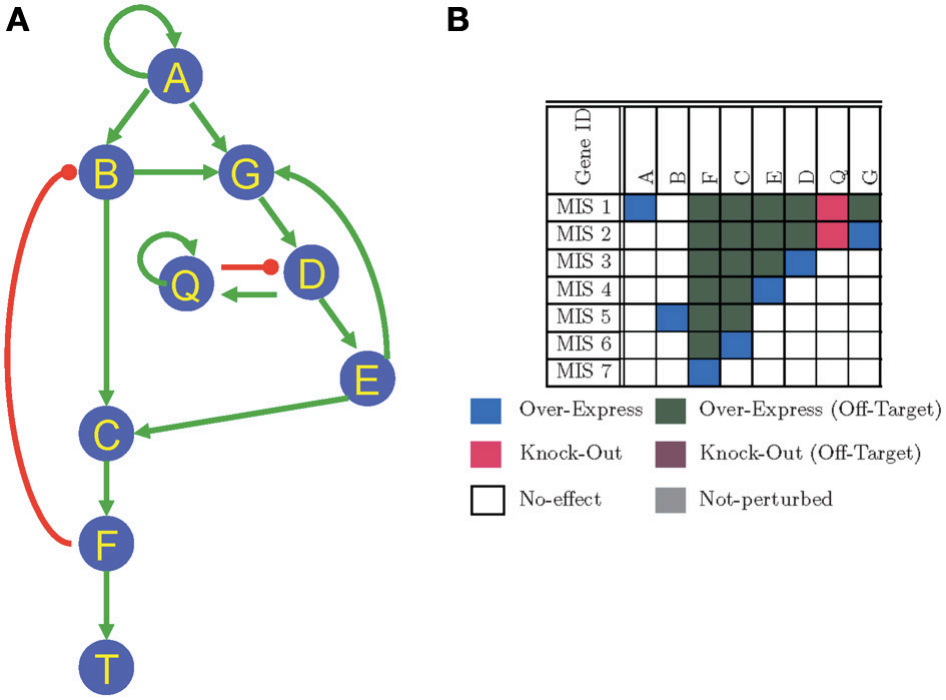
**(A)** A toy GRN representing interactions between a set of genes/proteins. Arrow-headed edges represent activation and circle-headed edges represent inhibiting interactions. **(B)** MIS patters to push the systems into a steady state where *T* = 1. For every MIS vector there is at least one mutations that has to be present. The polarity of these mandatory mutations are indicated by red (knock-out) or blue (over-express) colors. In addition, compatible off-target mutations and their polarities (over-expression or knock-out) are also listed for every MIS vector. Genes correponding to white color in an MIS vector indicate the mutations in these genes have no effect on the corresponding MIS vector. Genes correponding to gray color in an MIS vector should not be perturbed in any polarity (either by knock-out or over-expression). Else the corresponding MIS vector may not be able to generate the desired steady state.

A GRN, such as the one in Figure 1A, can be modeled as a Boolean network using the mapping we have introduced earlier in Garg et al. (2009). In the Boolean modeling of a GRN, a node can exist in two expression states: low and high. These two expression states are represented by logic 0 and 1, respectively. A state of the network at a given time instant *t* is defined by the expression state of all the nodes at that instant of time. A state of the network evolves over time as defined by the underlying interactions and stabilizes into a steady state or an attractor. An attractor is a set of states, such that once the network simulation reaches into one of the states of the attractor, it can only transition among the states within that attractor. An attractor with only a single state is referred to as a steady state. Here, we use the words attractors and steady states in the similar context of terminal states of the network simulations. Attractors of Boolean networks have been shown to correspond to the cellular phenotypes for various GRNs in the past (Kauffman, 1969; Mendoza and Alvarez-Buylla, 1998; Mendoza et al., 1999; Huang et al., 2005; Mendoza, 2005; Fauré et al., 2006; Klamt et al., 2006; Davidich and Bornholdt, 2008). Also it has been widely shown how the impact of gene perturbations can be mapped to experimental outcomes in terms of the steady states. Introducing a gene perturbation in a GRN, wherein a node is either over-expressed (i.e., constantly high expression state) or knocked-down (i.e., constantly low expression state) can change the steady states of the GRN and even push the system from one steady state into another steady state. Modeling the system-wide behavior of a GRN in terms of transition from one steady state to another in response to small perturbations can be used to systematically discover combinations of perturbations or MIS that can be of therapeutical advantage.

An MIS pattern gives the minimal possible combinations of gene perturbations required to force the GRN into a desired steady state and the term *minimal* in MIS implies that any other sub-set of an MIS pattern can not result in the same steady state behavior. However, more than one MIS patterns can generate the same steady state and for many practical applications, such as for screening a library of drug compounds, it would be necessary to enumerate a large number of such MIS patterns. An example of a list of MIS patterns that can force the GRN in Figure 1A into a steady state where the node *T* is at a high expression level (i.e., *T* = 1) are listed in Figure 1B. Although, most MIS patterns may seem trivial and involve regulating the direct upstream nodes of the node *T*, some non-trivial MIS patterns such as MIS 1 and MIS 2 may exist. The pattern MIS 1 states that the node *Q* should be knocked-out and node *A* should be over-expressed for forcing the GRN in Figure 1A into a steady state where *T* = 1. Such non-trivial MIS patterns that require multiple simultaneous perturbations are of utmost interest to algorithms developed in this manuscript.

The MIS problem has been addressed relatively sparingly in the past. Two recently published manuscripts by (Karlebach and Shamir, 2010; Samaga et al., 2010) propose algorithms to compute such minimal sets of targets in a given GRN. However, the algorithms proposed in both the manuscripts do not scale well with the size of the signaling pathway when it is desirable to enumerate MIS that would require more than three genes/proteins in the pathway to be simultaneously intervened. Moreover, these algorithms are not suitable for computationally screening drug compounds against pathways, as it is very rare to find drug compounds that would specifically target only a small list of genes/proteins highlighted in a given MIS. Therefore, in order to effectively screen drug compounds against pathways, it is necessary to also report compatible off-target genes/proteins along with every MIS of genes/proteins. Enumeration of compatible off-targets along with MIS has been completely disregarded in the previous works, rendering those algorithms of less practical significance.

The MIS algorithms proposed in this manuscript also report a list of compatible off-targets along with every minimal intervention set. We demonstrate the advantages of reporting off-targets by applying our algorithms on a database of miRNAs and their corresponding gene targets in order to discover disregulated miRNAs in cancer pathways. Our algorithm can also find many MIS, which requires taking into account the initial state of the pathway that corresponds to the resting state of the cell when the gene perturbation should be applied experimentally. We demonstrate the importance of taking into account the initial expression state of the GRN by taking the example of T-Helper GRN. We show that many therapeutically interesting MIS patterns for pushing the T-Helper cells into Th1 phenotype can be discovered only when the initial state of the T-helper GRN is set into the Th0 steady state.

## 2. Methods

Algorithms 1–4, presented in Figures 5, 6, describe the methodology followed to find a list of MIS patterns, given a GRN *G*, a target node *T* (which summarizes the desired steady state), and Boolean variable *outPolarity* (which specifies the desired polarity of the target node, i.e., 0 or 1). The function *comp_MIS()* as described in Algorithm 1 is the core function of our MIS generation methodology. The function *comp_MIS()* starts by unrolling the GRN into a tree-like structure starting from the target node *T* which has a fixed *polarity* (i.e., either high or low expression) in the desired final steady state (Line 3 of Algorithm 1). These nodes with fixed polarities are referred to as the root nodes of the network.

**Figure 2:**
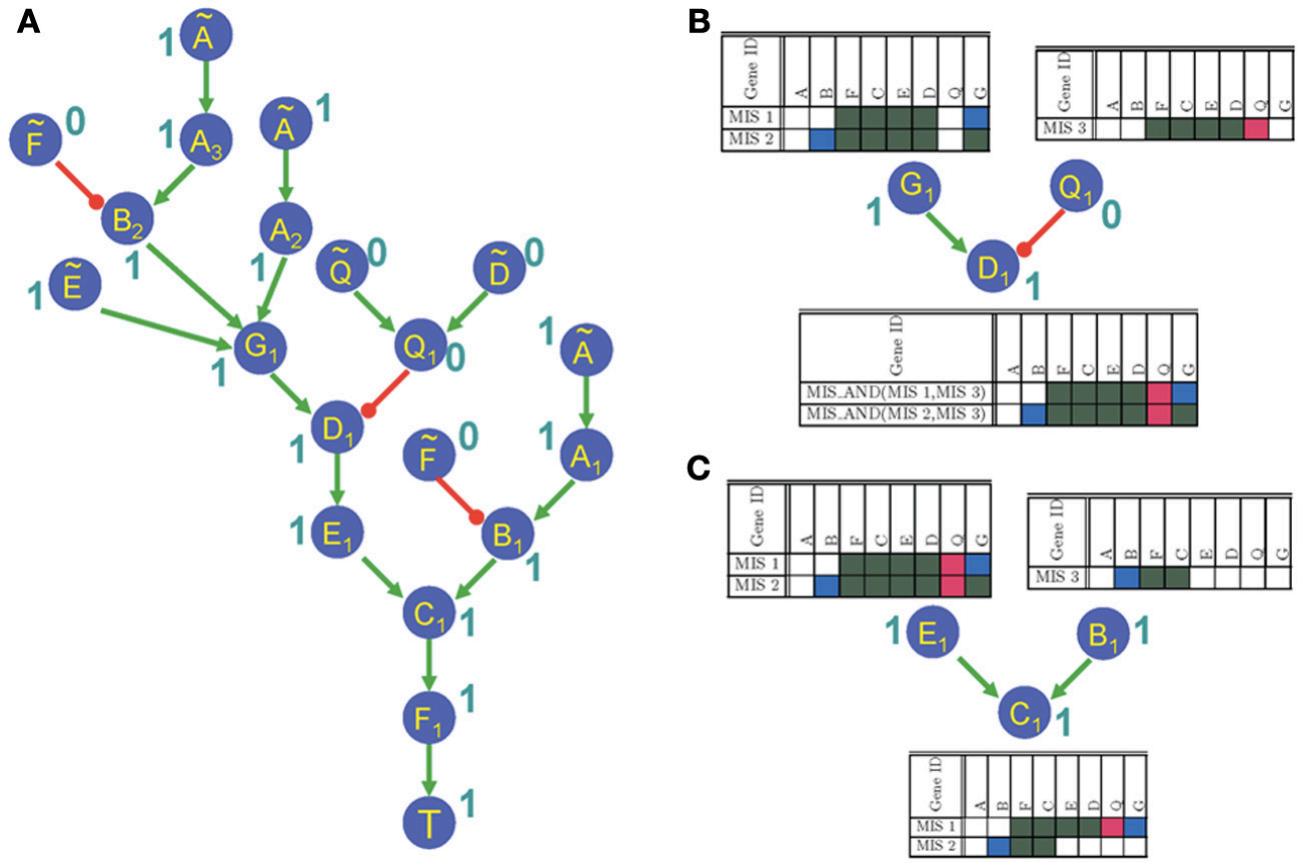
**(A)** Unrolled GRN when the node *T* has a fixed polarity in steady states. The labels 0 and 1 next to node labels represent polarity propagated from the root node *T* = 1 to the leaf nodes in the unrolled networks. **(B)** Propagation of MIS patterns through an AND gate. **(C)** Propagation of MIS patterns through an OR gate.

Algorithm 2 describes the GRN unrolling function *UnrollGRN()*. The function *UnrollGRN()* is called recursively starting from the root node (or target node) *T*. The GRN is unrolled along a path until a duplicate node is found. At that instance, if all the input nodes to the duplicate node already exist on the unrolled path, then the duplicate node is assigned a new name (with the symbol “~” over the original node name) and the unrolling process is terminated along this path. Otherwise, the duplicate node is further unrolled until the criteria for terminating the unrolling process is met. In each recursion, a set of nodes on the current path is maintained in the set of already unrolled nodes (labeled as *aN*). The current node being unrolled is added to set *aN* in Line 5 of Algorithm 2 and the input nodes (labeled as *g*) to the current node being unrolled (labeled as *S_n_*) are checked for duplicacy in Line 7. If the input node *g* does not exist in the set *aN*, then the *UnrollGRN()* process is repeated for that input node. In Lines 11–13, we check if any of the input nodes ĝ to the node *g* does not belong to the set of duplicate nodes *aN*, in which case the node *g* is further unrolled (Line 15). Otherwise the unrolling process is terminated along the current path by assigning the node ID corresponding to initial state nodes. The *orgIndex* vector maintains the index ID of all the nodes in the input GRN. The *UnrollGRN()* process returns a new node ID corresponding to unrolled node counterpart of original node *S_n_*. The new node IDs are generated by incrementing the counter corresponding to original set of nodes in the GRN in the vector *indexCount* (Line 18 of Algorithm 2). Vector *indexCount* maintains a count of the number of times a given node in the GRN is duplicated in the unrolling process, and helps ensuring that no two nodes have the same node ID in the unrolled GRN. The unrolled function with new unrolled node IDs corresponding to input nodes to node *S_n_* and new index of *S_n_* (i.e., *outIndex*) is generated in the function *composeFunc()* (described in Lines 24–30 of Algorithm 2). The *composeFunc()* duplicates the Boolean function describing the node *S_n_* in the original GRN and renames the input and output node with new node IDs that are generated in the function *UnrollGRN()*. An *offTargetIndex* (in Line 20 of Algorithm 2) is a vector of the set of nodes that contains all nodes that lie in between the current node being unrolled *S_n_* and the root node *T* of the unrolled GRN. The set of nodes in the *offTargetIndex* are used for specifying compatible off-targets corresponding to each MIS in Algorithm 3. The unrolled network generated by applying the *UnrollGRN()* function on the GRN in Figure 1A is shown in Figure 2A.

Once the input GRN is unrolled, Algorithm 1 generates the MIS vectors by recursively calling the function *genMIS()* starting from the unrolled index of target node *T* in Line 5 of Algorithm 1. The *genMIS()* is described in detail in Algorithm 3. The MIS vectors are generated by scanning the unrolled network from the root node (i.e., target node *T*) toward the leaf nodes. The function *genMIS()* is called recursively for the inputs of a given node (specified by *currNode* in Algorithm 3). The MIS vectors corresponding to input nodes are merged as described in the function *mergeMIS()*. The merged MIS vectors are combined with the new MIS vector which is generated for the given *currNode* using the function *newMIS()*. The *mergeMIS()* function as described in Algorithm 3, starts by setting Boolean variable *flagOR* to *TRUE* for Boolean input-output relationships that should be treated as an OR function (Lines 11–17). If the *flagOR* variable is *TRUE*, then the MIS vectors corresponding to input nodes to *currNode* are merged using the *MIS_OR* function (as decribed in Algorithm 4). Otherwise the input MIS vectors are merged using the *MIS*_*AND* function. The *newMIS()* function, generates a new MIS vector for the *currNode*. It returns a Boolean vector of length six times the number of number of nodes in the original GRN. The Boolean vector can be divided into three fragments of equal number of bits. The first fragment stores the information corresponding to *offTargetIndex* nodes, the second fragment stores the information of nodes that should have a specific initial state (i.e., nodes with the symbol “~”), and the last fragment stores the information regarding the nodes that should be perturbed (i.e., over-expressed or knocked-out). Figure 2A shows the polarity of each node in the unrolled GRN as a result of applying the *genMIS()* function on the unrolled network.

The *MIS_OR()* and *MIS_AND()* are the core functions of our MIS algorithm and ensure *minimal* and *non-conflict* properties of generated MIS vectors. Functions *MIS_OR()* and *MIS_AND()* merge the list of MIS vectors in a Boolean OR or AND manner as described in Algorithm 4. The *MIS_OR()* function checks for setwise containment of an MIS vector from one input list in another input list (Lines 5 and 7) and maintains the minimal property of generated MIS vectors by dropping MIS patterns that are not minimal in Lines 6 and 8 of Algorithm 4. The *MIS_AND()* function, checks for conflict between the MIS vectors in two input lists (Lines 23–25). Before merging any two MIS vectors, it checks if the resulting MIS vector would give rise to a conflicting perturbations (i.e., the same node over-expressed in one input MIS and knocked-out in the other MIS). Any such MIS vectors are dropped from the merged list of two input MIS vectors. Small examples further demonstrating the *MIS_AND()* and *MIS_OR()* functions are described in Figures 2B,C, respectively. In Figure 2B, the MIS pattern at the input node *E*_*1*_, which requires two perturbations involving over-expression of node *B* and knock-down of node *Q* is dropped from the merged MIS list at the output, as it is not a minimal pattern and can be replaced by the minimal MIS at the input node *B*_*1*_, which only requires one perturbation involving over-expression of node *B*.

Function *genMIS()* in Line 5 of Algorithm 1 returns minimal perturbation sets obtained by traversing the unrolled GRN. However, presence of feedback loops may prevent some of these MIS patterns from generating the desired steady state in actual simulations. Therefore, in order to ensure that the generated MIS can indeed give rise to desired steady state, we simulate the generated MIS patterns using the the function *simulateMIS()*, which essentially uses the Algorithm we developed in Garg et al. (2008) to model gene perturbations and checks if the resulting steady state are indeed the ones that are expected from the *comp_MIS()* function. The *simulateMIS()* function also checks for minimality of generated MIS patterns. These two additional steps, ensure that the list of MIS patterns resulting from *comp_MIS()* are indeed a minimal list of perturbed genes that can push the system into a desired cellular phenotype.

## 3. Results

We show the application of our algorithms on T-Helper cell and Growth vs. Apoptosis gene regulatory networks.

### 3.1. T-helper GRN

**Figure 3:**
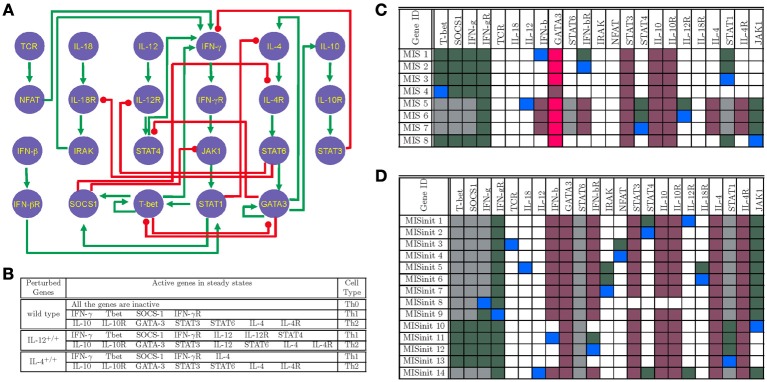
**(A)** T-Helper Gene Regulatory Network. Green arrow-headed edges represent activating interactions and red colored round-headed edges represent inhibiting edges. **(B)** Profile of three steady states present in T-Helper GRN representing Th0, Th1, and Th2 cellular phenotypes. **(C)** MIS patterns indicating gene-perturbations in-order to generate Th1 steady states. These MIS patterns have no pre-requirement on the initial state of the network. **(D)** MIS patterns to push the system into Th1 phenotype when the system is initially in Th0 steady state.

We have previously demonstrated existence of three steady states in a Boolean model of the T-helper GRN [Figures 3A,B, (Garg et al., 2008)]. These three steady states correspond to molecular profiles observed in Th0, Th1, and Th2 cells, which can be distinguished at the molecular level by their pattern of cytokine secretion that play a central role in cell mediated immunity (Th1 cells) and humoral responses (Th2 cells).

When we applied our MIS algorithm on the GRN of Figure 3A, it reported 22 MIS patterns that can drive the Th0 steady state into Th1 steady state. Out of these 22 MIS patterns, 8 patterns (Figure 3C) do not have any pre-requirement on initial state of the network and can lead to Th1 steady state independent of the initial state of the network. The remaining 14 MIS (Figure 3D) would lead to Th1 steady state only if the network is initially in the Th0 state. It is interesting to note that all the MIS patterns are composed of either a single or at most two simultaenous perturbations. Most of the gene perturbation combinations among the 14 MIS patterns in Figure 3D are well-known gene perturbations for biasing the T-Helper cellular differentiation toward Th1 cell types and have been used as target for well-known drug molecules to enhance immune response. The existing methods to find MIS patterns, which do not take into account initial state of the network, will only be able to identify 8 MIS patterns listed in Figure 3C. All these 8 MIS patterns require either T-bet over-expression or GATA3 knock-down in combination with few other perturbations. However, both T-bet and GATA3 are known to be difficult to target using drug compounds and can only be supressed or activated by using siRNAs or through indirect stimulation of upstream cytokines (Weigmann and Neurath, 2002; Usui et al., 2003; Liberman et al., 2009, 2012; Chou et al., 2010). More interesting perturbations identified by the remaining 14 MIS patterns in Figure 3D highlight the importance of considering initial state of the GRN in the algorithms presented in this manuscript.

The Th1 cells produce IFNg as their signature cytokine secretion profile. Secreted IFNg can bind to its receptor IFNgR present on the Th1 cell surface leading to activation of signaling pathways involved in Th1 cell differentiation and maintenance of Th1 state (Novelli et al., 1997; Murphy and Reiner, 2002). The MIS patterns, *MISInit 8* and *9*, in Figure 3D represent over-expression of IFNg or IFNgR, respectively, that are required for differentiation of Th0 cells to Th1 phenotype. The MIS pattern *MISInit 3* represents activation of T cell receptor (TCR) through external ligands, which can lead to transcriptional expression of various cytokines involved in T cell differentiation. The remaining MIS patterns represent other well-known modes of differentiating Th0 to Th1 cell types either through production of IFNg in T helper cells or by activating proteins downstream of IFNg signaling (Mendoza, 2005; Mendoza and Xenarios, 2006). These modes of activation include expression of external ligands such as IL-18, IL-12, and IFN-b (*MISInit 5*, *11* and *14*), or by directly stimulating their target receptors IL-18R, IL-12R, and IFN-bR (*MISInit 6*, *1* and *12*), increased expression of intermediate Kinases IRAK and JAK1 (*MISInit 7* and *10*), or by expressing transcription factors STAT1, STAT4, or NFAT (*MISInit 13*, *2* and *4*). The compatible off-target perturbations in the MIS patterns listed in Figure 3D, are mostly related to down-regulating the expression of ligands and other proteins involved in maintenance of Th2 cell type, and differentiation of Th0 to Th2 cell types (Mendoza, 2005; Mendoza and Xenarios, 2006).

### 3.2. Growth vs. apoptosis GRN

**Figure 4:**
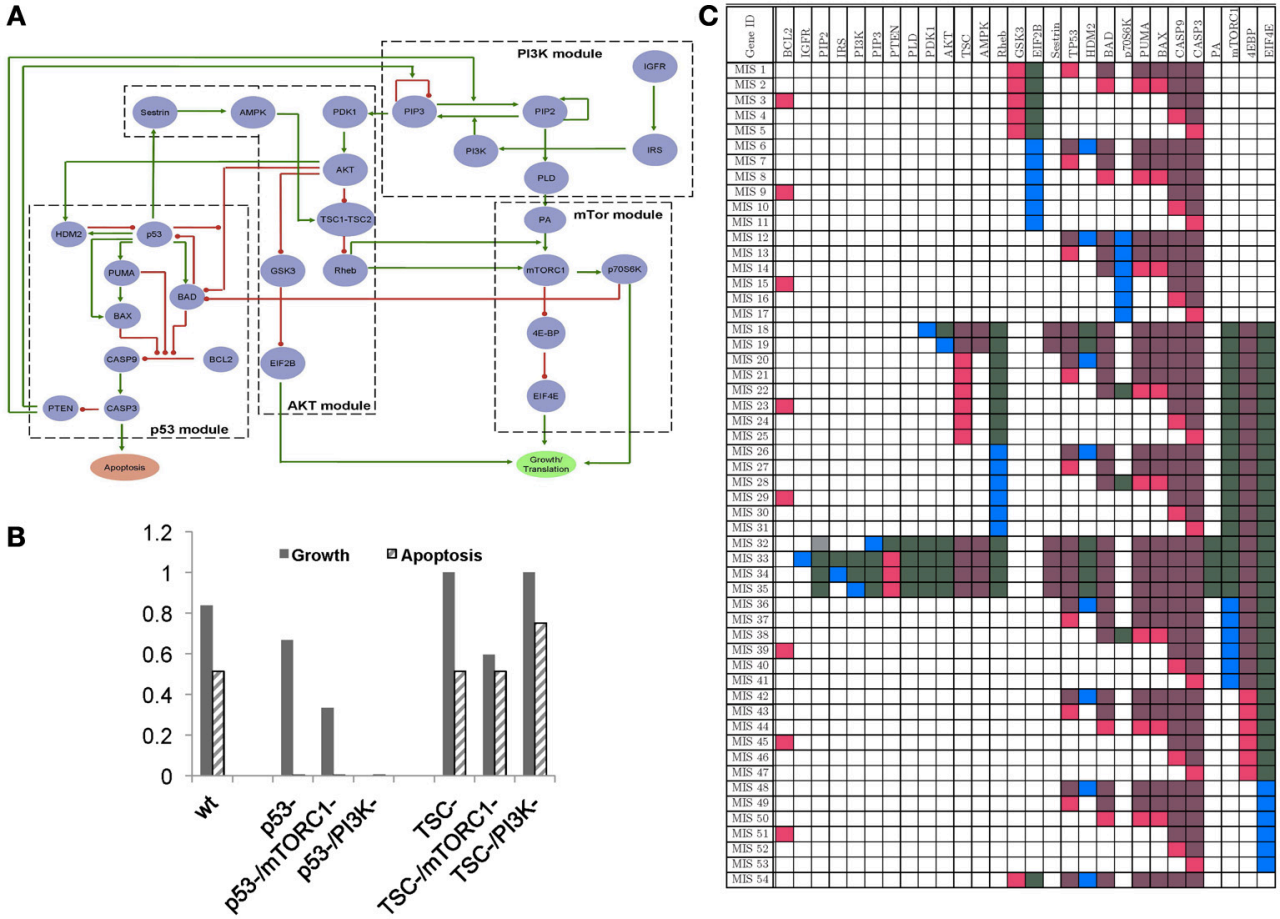
**(A)** GRN representing interactions among some proteins known to play a crucial role in maintaining a balance between apoptosis and cellular growth in cancer signaling pathways. **(B)** Distribution of growth and apoptosis signals in the steady states of the wild-type GRN [labeled (wt)], or in the presence of p53 or TSC knock-out mutations (labeled p53- and TSC-), and of the GRN where the knock-out affects of drugs targeting mTORC1 (labeled mTORC1-) and PI3K (labeled PI3K-) are modeled. **(C)** MIS patterns indicating gene-perturbations necessary for in-order to push the system into the state correponding to constitutive high-growth and low-apoptosis signals.

**Figure 5:**
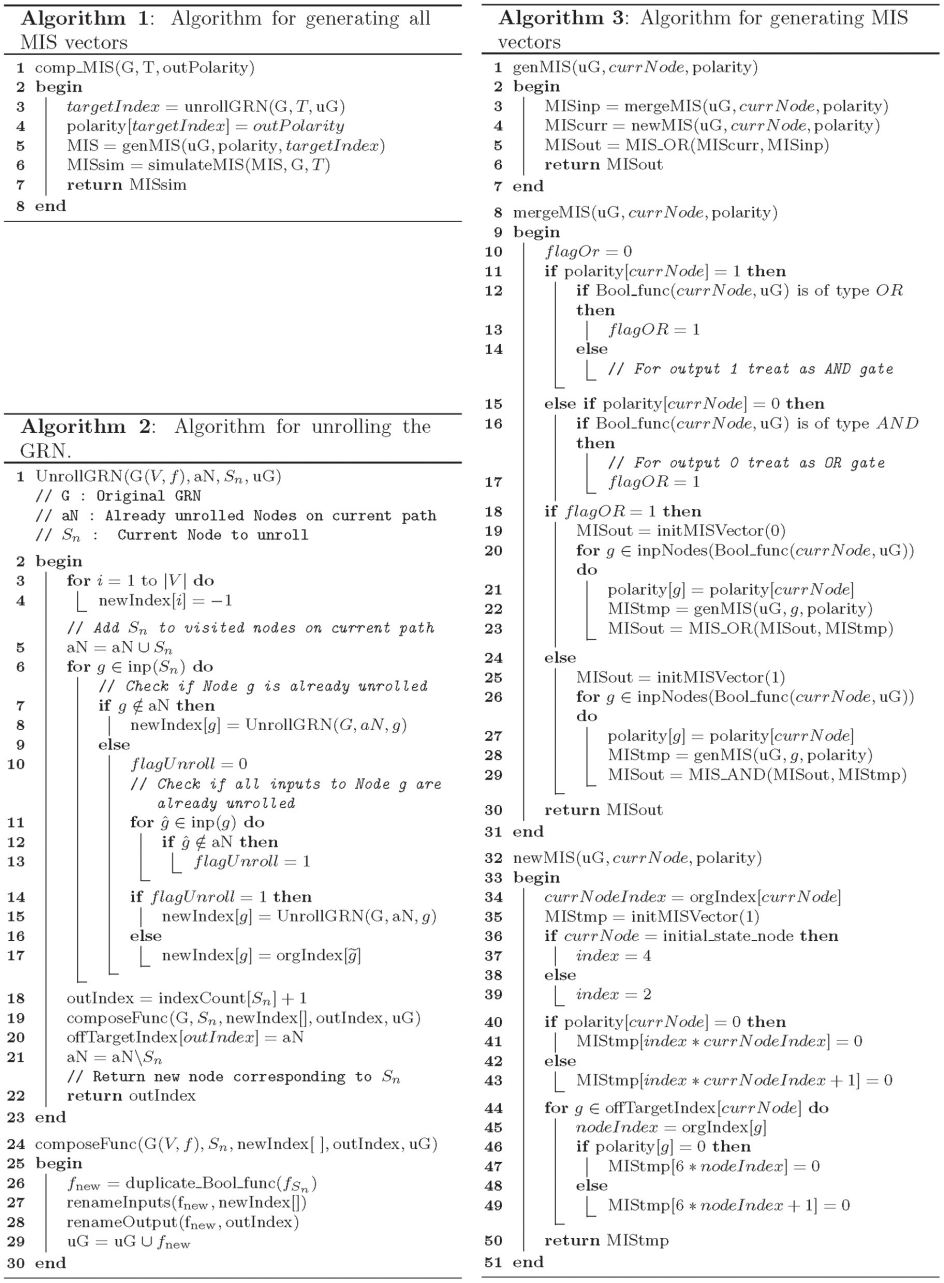
**Algorithms for computing the MIS patterns given a Boolean GRN and the profile of desired steady states**.

We identified key proteins that are known to play a crucial role in maintaining the balance between growth and apoptosis signals in the cancer pathways. We then constructed the GRN representing interactions between these proteins by identifying experimentally validated functions defining these interactions from the literature. Boolean formulation of these interactions are summarized in the GRN in Figure 4A Figure 5 and literature evidence supporting these interactions are listed in Supplementary Table 1. The GRN can be divided among four modules—PI3K, AKT, p53, and mTORC1—to represent four sub-networks that are mostly comprised of linear paths (or no feedback loops). These four modules interact with each other through positive and negative interactions giving rise to multiple feedback loops in the resulting GRN. As a result of these feedback loops, the GRN in Figure 4A gives rise to four Boolean attractors when simulated using our Boolean modeling toolbox genYsis (Garg et al., 2008). Both the attractors show oscillating apoptosis and growth signals demonstrating the ability of the pathway to self-regulate the cellular growth. Figure 4B shows the average growth and apoptosis signals in these attractors. An equal distribution of states displaying high growth and high apoptosis in these Boolean attractors highlight the balance maintained by the feedback loops in the constructed GRN.

**Figure 6:**
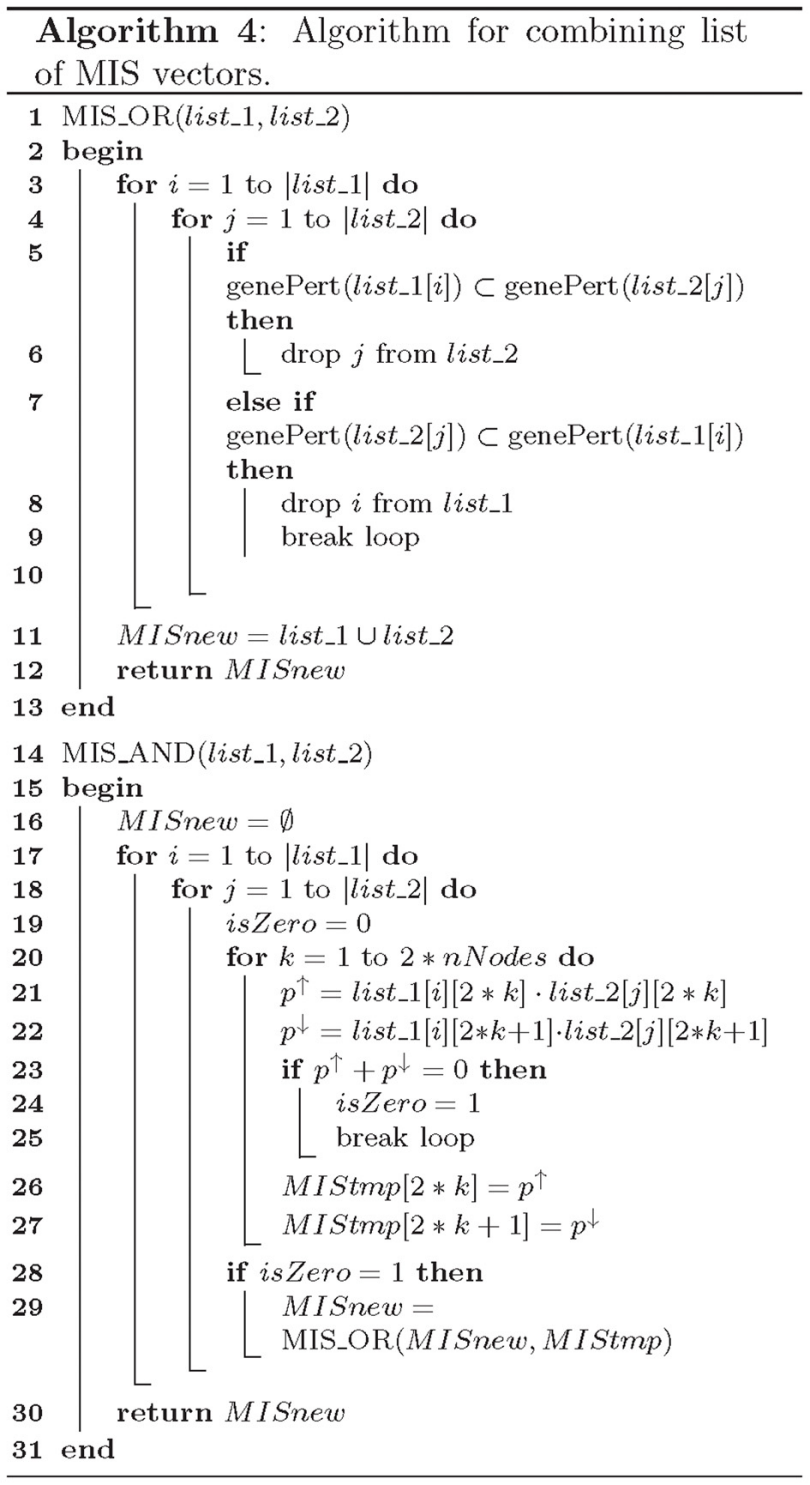
**Algorithms for merging MIS patterns**.

We modeled the impact of two well-known cancer mutations in genes p53 and TSC2 on the attractors of GRN in Figure 4A. The p53 gene is one of the most frequently mutated genes in human cancers (Bourdon, 2007; Vousden and Lane, 2007). The p53 mutation leads to constitutive inhibition of its functionality and suppresses apopotosis signals, leading to uncontrolled cell growth or proliferation. Our simulation results also capture this effect of p53 mutation, wherein the average apoptosis signals in the attractor states decreases to 0 and growth signals stay high as compared to attractors in wild-type GRN (Figure 4B). We also modeled the impact of mTORC1 and PI3K inhibition (representing the affect of drug compounds such as Rapamycin and Wortmannin, respectively) on restoring the balance of growth vs. apoptosis in the presence of p53 mutation (Figure 4B). From the simulation results in Figure 4B, it is clear that while PI3K inhibition can balance the p53 mutation by completely suppressing growth signals, mTORC1 inhibition can only moderately decrease the constitutive growth signals resulting from p53 mutations. This can be attributed to PI3K inhibitor acting upstream of mTORC1 in order to block all the three distinct pathways leading to cell division and protein production (Welsh et al., 1997; Mendez et al., 2001; Liang et al., 2003). On the contrary, in the presence of mutation in TSC2 gene (leading to loss of function of TSC2 and high growth vs. apoptosis signals in attractor states), simulation results show that growth signals cannot be regulated with PI3K inhibitors (Figure 4B). However, mTORC1 inhibitor can counter the effect of TSC2 mutation by restoring the growth signals to low level of wild-type steady state levels. It has been shown in the experiments that cells treated with PI3K inhibitors show improved apoptosis response in a TSC2 expression dependent manner (Kolb et al., 2005). On the other hand, greatly elevated mTORC1 signaling has been reported in the presence of TSC2 mutation, which can be restored with mTORC1 inhibitors (Kim et al., 2011).

Having established a confidence in our constructed GRN for modeling the balance between growth and apoptosis signals in cancer pathways, we next computed MIS patterns that can force the dynamics of the network to constitutive high growth and constitutive low apoptosis signals. Our algorithm reported a total of 54 MIS patterns, none of which requires the system to be in a specific initial state (Figure 4C). Most of the perturbations (over-expression or knock-out) defined by these MIS patterns are well-known mutations in cancer cells. Most of the MIS patterns listed in Figure 4C require two or more simultaneous perturbations, highlighting that highly malignant cell phenotypes often comprise of multiple mutations. For example, MIS patterns that contain either p53 or TSC mutations also require mutations in other parts of the pathway to push the system into desired steady state. This observation is also supported from the simulation results in Figure 4B, where these mutations alone cannot give rise to constitutively high growth and low apoptosis signals in steady states. Another perturbation vector given by MIS 19 represents the over-activated AKT protein, which may explain a well-known mutation in AKT that leads to hyper-phosphorylated form of AKT in cancer cells.

We next screened these MIS patterns against a database of miRNA and their target gene pairs in human genome, which we compiled using four different miRNA target prediction algorithms [miRANDA (John et al., 2004), TargetScan (Lewis et al., 2005), mirDB (Wang and El Naqa, 2008) and RNA22 (Miranda et al., 2006)] to predict miRNA-target pairs and combined these with experimentally validated pairs [TarBase, (Papadopoulos et al, 2009)]. *In silico* miRNA target prediction algorithms suffer high rates of type I and type II errors (Watanabe et al., 2007; Zhang and Verbeek, 2010). Therefore, in order to increase the quality of the predictions we considered only those predictions that were supported by at least two different prediction algorithms, or were experimentally validated. We restricted the application of our algorithm to 464 miRNA cancer type pairs that were collected from different experimental studies in literature and have been shown to be disregulated (either up-regulated or down-regulated) in various different human cancers or cancer cell lines (Sinha et al., 2008).

If a given miRNA targets a gene identified as over-expressed in an MIS pattern, then we define the polarity of dis-regulation of that miRNA as down-regulated. Similarly, a given miRNA is said to be up-regulated if its target gene is knocked-down in the MIS pattern. When disregulation of only a single miRNA is assumed to lead to cancerous behavior (i.e., only looking at MIS patterns with all knock-down genes or all over-expressed genes), our algorithm predicts 33 up-regulated and 20 down-regulated miRNAs. The predicted miRNAs, their polarity, corresponding cancer types and supporting MIS patterns are listed in Supplementary Table 2. Out of these predicted miRNAs, 38 miRNAs were found to have the same polarity as has been seen in published experimental results and 15 predicted miRNAs did not match the polarity observed in published experiments (Sinha et al., 2008). However, we believe that the small list of miRNA with mismatched polarity may arise from the fact that two or more miRNA may be simultaneously perturbed in the cancer types studied in published results and require analyzing the effect of disregulation in more than one miRNA along with our list of MIS patterns.

## 4. Discussion

In this manuscript, we have presented an efficient approach to generate a list of minimal sets of gene perturbations that can push the dynamics of GRN into a specific steady state (representing a given cellular phenotype). Our algorithm for computing MIS patterns follows a branch and bound approach, where the unrolled network is scanned for MIS patterns in a depth-first manner. If the network being simulated has many feedback loops, then the current implementation of Algorithms proposed in this manuscript may be inefficient due to the large size of the unrolled network (Supplementary Table 3). However, the unrolled network can be scanned for MIS patterns in an efficient manner by a parallel implementation of the function *genMIS()* described in Algorithm 3. The efficiency of MIS computation can be further enhanced by parallelizing the function *simulateMIS()*, which simulates and tests the minimality of the MIS patterns generated by the function *genMIS()*.

The algorithms proposed here can be useful in various experimental settings where one would like to enumerate multiple options to regulate the dynamics of GRNs. We demonstrate one such application of our algorithm with respect to T-Helper GRN. The T-helper GRN shown in Figure 3A has been previously shown to effectively model the precursor Th0 cells and effector Th1 and Th2 cells (Mendoza and Xenarios, 2006). Understanding the molecular mechanisms that regulate the differentiation process from Th0 toward either Th1 or Th2 is very important, since an immune response biased toward the Th1 phenotype result in the appearance of autoimmune diseases, and an enhanced Th2 response can originate allergic reactions (Murphy and Reiner, 2002; Agnello et al, 2003). We previously demonstrated how one can efficiently simulate the effect of gene perturbations in the T-Helper GRN (Garg et al., 2008, 2009). However, it would be of utmost interest to generate a list of possible perturbations that can transition a cell from one steady state to another. For example, MIS patterns required to transition a cell from Th0 to Th1 cell state would indicate different possible treatments to stimulate the auto-immune response of the body. The MIS patterns generated by our algorithm captures some of the well-known perturbations that have been experimentally validated to differentiate T-Helper cells into Th1 phenotype. Gene expression levels can often exist at more than two expression states. The algorithms proposed here can be extended to multiple expression levels of nodes (such as Low/Medium/High) by encoding them into Boolean rules (Garg et al., 2007).

The GRN presented here for modeling the growth vs. apoptosis signals in cancer pathways, consists of various feedback loops that ensures the balance between the growth and apoptosis pathways. It is a well-accepted fact that mutations leading to permanent loss or gain of function of genes in the feedback loops of signaling pathways can disregulate the delicate balance between pro-growth and pro-apoptopic cellular signals. If these mutations are in a favor of pro-growth or anti-apoptosis signals, then a cell is said to have a pre-disposition toward uncontrolled growth (and hence proliferation). In such a scenario, a cell (carrying mutations) undergoes uncontrolled proliferation and can subsequently lead to the formation of tumors. Most cancer therapies either try to restore the normal expression of mutated genes directly or counteract the impact of mutated genes by targeting other genes (or proteins) in the pathway. Understanding how different genes (and proteins) regulate each other in these pathways is therefore of major interest in the development of treatments for various cancers. Here, we show how the impact of such mutations can be studied with respect to our proposed GRN by modeling p53 and TSC mutations. In Figure 4B, differential response of therapies targeting PI3K and mTORC1 nodes in the presence of p53 and TSC mutations indicate the importance of taking into account gene mutations when deciding upon the drug therapies. Whereas, only single mutations are simulated in Figure 4B, in a real world scenario, multiple mutations can be present simultaneously. In such a scenario, activity of multiple genes/proteins may have to be targeted for the drug therapy to be effective. Such compatible sets of genes/proteins which would be suitable for manipulation by one or more drug compounds can be quickly discovered by enumerating all MIS vectors of a GRN. Here, we demonstrate one such application by listing MIS vectors that can lead to high growth and low apoptosis signals in steady state of GRN of Figure 4A.

We also demonstrate an example of how the MIS patterns generated by our algorithm could be used to predict miRNAs which, when over or under-expressed, could lead to a cancerous phenotype of cells. Aberrant expression of miRNAs is known to play a major role in the development of cancers due to their importance in various biological processes such as cellular proliferation and apoptosis (Subramanian and Steer, 2010; Yu et al., 2010). However, despite much interest in this area, the mechanism of action of miRNAs in disease remains largely unknown. The current view is that miRNAs have evolved to coordinately regulate cellular processes; thus, whilst the number of miRNAs is relatively small compared to the number of genes [<1000 in humans, (Griffiths-Jones et al., 2008)], complex regulation mechanisms can be achieved through the combined actions of multiple miRNAs acting in a temporal and spatial manner. In our analysis, of 53 miRNAs predicted to influence the cancerous phenotype, 38 (~71%) show the same polarity as published data, demonstrating the potential of this approach. It remains to be seen whether the remaining 15 predicted miRNAs that do not show the same polarity as published data, have an impact on defining the cancerous phenotype.

In addition to applications shown in this manuscript, such as discovering minimal functional mutations in a disease phenotype and screening drug compound library, one can use the MIS vectors to guide experimental setups. Normally, in the absence of information of MIS patterns, one would have to try all possible combinations (*3*^*N*^) of *N* different perturbations (compounds or treatments) of genes/proteins in a given signaling pathway, which can quickly lead to a large number of experiments for even a modestly large GRN. Even when number of simultaneous perturbations to test are restricted to two, it becomes unfeasible to perform all possible two-combinations of available compounds. In such a scenario, MIS patterns generated by our algorithm can be very useful. The number of MIS patterns that can lead to a desired steady state can be significantly smaller than the number of all possible perturbations as has been seen from the application of our algorithm on the T-Helper and Apoptosis vs. Growth GRNs (Figures 3, 4 and Supplementary Table 3). Generating such MIS patterns algorithmically using an approach presented here can therefore reduce both the number of simulations and experiments required, and can provide model-driven insight into which subsets of genes should be knocked-down and over-expressed to obtain a desired cellular phenotype.

### Conflict of interest statement

The authors declare that the research was conducted in the absence of any commercial or financial relationships that could be construed as a potential conflict of interest.

## Supplementary material

Supplementary Table 1 **List of Boolean interactions in the Growth vs. Apoptosis GRN along with supporting references**.

Supplementary Table 2 **List of predicted miRNA with matching (and mis-matching) polarity, Cancer types, and supporting MIS IDs**.

Supplementary Table 3 **Simulation results of computing MIS patterns on Random Networks of varying complexities**.

## Research Topic:

From structural to molecular systems biology: experimental and computational approaches to unravel mechanisms of kinase activity regulation in cancer and neurodegeneration

## Topic Editor:

Matteo Barberis, Humboldt University Berlin, Germany; Max Planck Institute for Molecular Genetics, Berlin, Germany
