# Supplementary material for: Efficient computation of minimal perturbation sets in gene regulatory networks
Source: Front Physiol. 2013 Dec 17;4:361. doi: 10.3389/fphys.2013.00361 (PMC3867968; doi:10.3389/fphys.2013.00361)
Supplement: Supplementary Table 1 — List of Boolean interactions in the Growth vs. Apoptosis GRN along with supporting references. [file DataSheet1.PDF]

Supplementary Table 1. List of Boolean interactions in the Growth vs. Apoptosis GRN (Figure 4) along with supporting references.

**Source**	**Interaction**	**Target**	**Reference**
4EBP	INHIBITION	EIF4E	Gingras et al., 1999; Shahbazian et al., 2006; Matsuo et al., 1997
AKT	INHIBITION	GSK3	Lee and Kim, 2007; Cohen and Frame, 2001
AKT	ACTIVATION	HDM2	Mayo and Donner, 2001
AKT&^AMPK	INHIBITION	TSC	Budanov and Karin, 2008; Benvenuto et al., 2000; Chong-Kopera et al., 2006
AKT&^TP53	INHIBITION	BAD	Jiang et al., 2006; Jiang et al., 2007; Datta et al., 1997
BAD	INHIBITION	TP53	Jiang et al., 2006; Jiang et al., 2007
BCL2&BAD	ACTIVATION	CASP9	Yu et al., 2001; Chipuk et al., 2004; Nakano and Vousden, 2001
BCL2&BAX	ACTIVATION	CASP9	Yu et al., 2001; Nakano and Vousden, 2001
BCL2&PUMA	ACTIVATION	CASP9	Yu et al., 2001; Nakano and Vousden, 2001
CASP3	ACTIVATION	Apoptosis	Zou et al., 2003
CASP3	INHIBITION	PTEN	Torres et al., 2003
CASP9	ACTIVATION	CASP3	Zou et al., 2003
EIF2B	ACTIVATION	Growth	Dever, 2002
EIF4E	ACTIVATION	Growth	Gingras et al., 1999; Shahbazian et al., 2006; Matsuo et al., 1997
GSK3	INHIBITION	EIF2B	Dever, 2002
HDM2	INHIBITION	TP53	Mayo and Donner, 2002
IGFR	ACTIVATION	IRS	Zha et al., 2010
IRS	ACTIVATION	PI3K	Lackey et al., 2007; Easton et al., 2006
mTORC1	INHIBITION	4EBP	Gingras et al., 1999; Shahbazian et al., 2006; Matsuo et al., 1997
mTORC1	ACTIVATION	p70S6K	Burnett et al., 1998
p70S6K	INHIBITION	BAD	Harada et al., 2001
p70S6K	ACTIVATION	Growth	Loreni et al., 2000; Harada et al., 2001; Heinonen et al., 2008; Jefferies et al., 1997; Burnett et al., 1998
PA&Rheb	ACTIVATION	mTORC1	Foster, 2007; Bai et al., 2007; Chen et al., 2005; Sun et al., 2008

PDK1	ACTIVATION	AKT	Alessi et al., 1996; Blume-Jensen and Hunter, 2001
PIP2	ACTIVATION	PIP2	Gervais et al., 2008
PIP2	ACTIVATION	PLD	Foster and Xu, 2003
PIP2&PI3K	ACTIVATION	PIP3	Toker and Cantley, 1997; Cully et al., 2006; Czech, 2000
PIP3	ACTIVATION	PDK1	Alessi et al., 1996; Blume-Jensen and Hunter, 2001
PIP3&PTEN	ACTIVATION	PIP2	Sun et al., 1999; Cully et al., 2006; Czech, 2000; Kim and Mak, 2006
PIP3&PTEN	INHIBITION	PIP3	Sun et al., 1999; Cully et al., 2006; Czech, 2000; Kim and Mak, 2006
PLD	ACTIVATION	PA	Foster and Xu, 2003
PUMA	ACTIVATION	BAX	Chipuk et al., 2004; Vogt et al., 2006
Rheb	ACTIVATION	mTORC1	Bai et al., 2007
Sestrin	ACTIVATION	AMPK	Budanov and Karin, 2008
TP53	ACTIVATION	BAD	Yu et al., 2001; Nakano and Vousden, 2001; Jiang et al., 2006; Jiang et al., 2007
TP53	ACTIVATION	BAX	Chipuk et al., 2004; Vogt et al., 2006
TP53	ACTIVATION	HDM2	Piette et al., 1997
TP53	ACTIVATION	PUMA	Yu et al., 2001; Nakano and Vousden, 2001
TP53	ACTIVATION	Sestrin	Peeters et al., 2003; Velasco-Miguel et al., 1999
TSC	INHIBITION	Rheb	Inoki et al., 2003; Saucedo et al., 2003

References

Alessi DR, Andjelkovic M, Caudwell B, Cron P, Morrice N, Cohen P, and Hemmings BA. Mechanism of activation of protein kinase B by insulin and IGF-1. EMBO Journal, 15:6541-6551, 1996.

Bai X, Ma D, Liu A, Shen X, Wang QJ, Liu Y, and Jiang Y. Rheb activates mTOR by antagonizing its endogenous inhibitor, FKBP38. Science, 318:977-980, 2007.

Benvenuto G, Li S, Brown SJ, Braverman R, Vass WC, Cheadle JP,Halley DJ, Sampson JR, Wienecke R, and DeClue JE. The tuberous sclerosis-1 (TSC1) gene product hamartin suppresses cell growth and augments the expression of the tsc2 product tuberin by inhibiting its ubiquitination. Oncogene, 19:6306-6316, 2000.

Blume-Jensen P and Hunter T. Oncogenic kinase signalling. Nature, 411:355-365, 2001.

Budanov AV and Karin M. p53 target genes sestrin1 and sestrin2 connect genotoxic stress and mTOR signaling. Cell, 134:451-460, 2008.

Burnett PE, Barrow RK, Cohen NA, Snyder SH, and Sabatini DM. RAFT1 phosphorylation of the translational regulators p70 S6 kinase and 4E-BP1. Proceedings of National Academy of Science of the USA, 95:1432-1437, 1998.

Chen Y, Rodrik V, and Foster DA. Alternative phospholipase D/mTOR survival signal in human breast cancer cells. Oncogene, 24:672-679, 2005.

Chong-Kopera H, Inoki K, Li Y, Zhu T, Garcia-Gonzalo FR, Rosa JL,and Guan KL. TSC1 stabilizes TSC2 by inhibiting the interaction between TSC2 and the HERC1 ubiquitin ligase. Journal of Biological Chemistry, 281:8313-8316, 2006.

Chipuk JE, Kuwana T, Bouchier-Hayes L, Droin NM, Newmeyer DD, Schuler M, and Green DR. Direct activation of Bax by p53 mediates mitochondrial membrane permeabilization and apoptosis. Science, 303:1010-1014, 2004.

Cohen P and Frame S. The renaissance of GSK3. Nature Reviews Molecular Cell Biology, 2:769-776, 2001.

Cully M, You H, Levine AJ, and Mak TW. Beyond PTEN mutations: the PI3K pathway as an integrator of multiple inputs during tumorigenesis. Nature Reviews Cancer, 6:184-192, 2006.

Czech MP. PIP2 and PIP3: complex roles at the cell surface. Cell, 100:603-606, 2000.

Datta SR, Dudek H, Tao X, Masters S, Fu H, Gotoh Y, and Greenberg ME. Akt phosphorylation of BAD couples survival signals to the cell intrinsic death machinery. Cell, 91:231-241, 1997.

Dever TE. Gene-specific regulation by general translation factors. Cell, 108:545-556, 2002.

Easton JB, Kurmasheva RT, and Houghton PJ. IRS-1: auditing the effectiveness of mTOR inhibitors. Cancer Cell, 9:153-155, 2006.

Foster DA. Regulation of mTOR by phosphatidic acid? Cancer Research, 67:1-4, 2007.

Foster DA and Xu L. Phospholipase D in cell proliferation and cancer. Molecular Cancer Research,1:789-800, 2003.

Gervais L, Claret S, Januschke J, Roth S, and Guichet A. PIP5K dependent production of PIP2 sustains microtubule organization to establish polarized transport in the drosophila oocyte. Development, 135:3829-3838, 2008.

Gingras AC, Gygi SP, Raught B, Polakiewicz RD, Abraham RT, Hoekstra MF, Aebersold R, and Sonenberg N. Regulation of 4E-BP1 phosphorylation: a novel two-step mechanism. Genes and Development, 13:1422-1437, 1999.

Harada H, Andersen JS, Mann M, Terada N, and Korsmeyer SJ. p70S6 kinase signals cell survival as well as growth, inactivating the pro-apoptotic molecule BAD. Proceedings of National Academy of Science of the USA, 98:9666-9670, 2001.

Heinonen H, Nieminen A, Saarela M, Kallioniemi A, Klefstrm J, Hautaniemi S, and Monni O. Deciphering downstream gene targets of PI3K/mTOR/p70S6K pathway in breast cancer. BMC Genomics, 9, 2008.

Inoki K, Li Y, Xu T, and Guan KL. Rheb GTPase is a direct target of TSC2 GAP activity and regulates mTOR signaling. Genes and Development, 17:1829-1834, 2003.

Jefferies HBJ, Fumagalli S, Dennis PB, Reinhard C, Pearson RB, and Thomas G. Rapamycin suppress 5'TOP mRNA translation through inhibition of p70s6k. EMBO Journal, 16:3693-3704,

1997.

Jiang P, Du W, Heese K, and Wu M. The Bad guy cooperates with a good cop p53: Bad is transcriptionally up-regulated by p53 and forms Bad/p53 complex at the mitochondria to induce apoptosis. Molecular and Cell Biology, 26:9071-9082, 2006.

Jiang P, Du W, and Wu M. p53 and Bad: remote strangers become close friends. Cell Research, 17:283-285, 2007.

Kim RH and Mak TW. Tumours and tremors: how PTEN regulation underlies both. British Journal of Cancer, 94:620-624, 2006.

Lackey J, Barnett J, Davidson L, Batty IH, Leslie NR, and DownesCP. Loss of PTEN selectively desensitizes upstream IGF1 and insulin signaling. Oncogene, 26:7132-7140, 2007.

Lee J and Kim MS. The role of GSK3 in glucose homeostasis and the development of insulin resistance. Diabetes research and Clinical Practice, 77:S49-S57, 2007.

Loreni F, Thomas G, and Amaldi F. Transcription inhibitors stimulate translation of 5' TOP mRNAs through activation of S6 kinase and the mTOR/FRAP signalling pathway. European Journal of Biochemistry, 267:6594-6601, 2000.

Mayo LD and Donner DB. A phosphatidylinositol 3-kinase/Akt pathway promotes translocation of Mdm2 from the cytoplasm to the nucleus. Proceedings of National Academy of Science of the USA, 98:11598-11603, 2001.

Mayo LD and Donner DB. p53-Mdm2-the affair that never ends. Carcinogenesis, 23:541-547, 2002.

Matsuo H, Li H, McGuire AM, Fletcher CM, Gingras AC, Sonenberg N, and Wagner G. Structure of translation factor ElF4E bound to m7GDP and interaction with 4E-binding protein. Natural Structural and Molecular Biology, 4:717-724, 1997.

Nakano K and Vousden KH. PUMA, a novel pro-apoptotic gene, is induced by p53. Molecular Cell, 7:683-694, 2001.

Peeters H, Debeer P, Bairoch A, Wilquet V, Huysmans C, Parthoens E, Fryns JP, Gewillig M, Nakamura Y, Niikawa N, Van de Ven W, and Devriendt K. PA26 is a candidate gene for heterotaxia in humans: identification of a novel PA26-related gene family in human and mouse. Human Genetics, 112:573-580, 2003.

Piette J, Neel H, and Marchal V. Mdm2: keeping p53 under control. Oncogene, 15:1001-1010, 1997.

Saucedo LJ, Gao X, Chiarelli DA, Li L, Pan D, and Edgar BA. Rheb promotes cell growth as a component of the insulin/TOR signalling network. Nature Cell Biology, 5:566-571, 2003.

Shahbazian D, Roux PP, Mieulet V, Cohen MS, Raught B, Taunton J, Hershey JWB, Blenis J, Pende M, and Sonenberg N. The mTOR/PI3K and MAPK pathways converge on eIF4B to control its phosphorylation and activity. EMBO Journal, 25:2781-2791, 2006.

Sun H, Lesche R, Li DM, Liliental J, Zhang H, Gao J, Gavrilova N, Mueller B, Liu X, and Wu H. PTEN modulates cell cycle progression and cell survival by regulating phosphatidylinositol 3,4,5,-trisphosphate and Akt/protein kinase B signaling pathway. Proceedings of National Academy of Science of the USA, 96:6199-6204, 1999.

Sun Y, Fang Y, Yoon MS, Zhang C, Roccio M, Zwartkruis FJ, Armstrong M, Brown HA, and Chen J. Phospholipase D1 is an effector of Rheb in the mTOR pathway. Proceedings of National Academy of Science of the USA, 105:8286-8291, 2008.

Toker A and Cantley LC. Signalling through the lipid products of phosphoinositide-3-OH kinase. Nature,387:673-676, 1997.

Torres J, Rodriguez J, Myers MP, Valiente M, Graves JD, Tonks NK, and Pulido R. Phosphorylation-regulated cleavage of the tumor suppressor PTEN by Caspase-3.. Journal of Biological Chemistry, 278: 30652-30660, 2003.

Velasco-Miguel S, Buckbinder L, Jean P, Gelbert L, Talbott R, Laidlaw J, Seizinger B, and Kley N. PA26, a novel target of the p53 tumor suppressor and member of the GADD family of DNA damage and growth arrest inducible genes. Oncogene, 18:127-137, 1999.

Vogt M, Butz K, Dymalla S, Semzow J, and Hoppe-Seyler F. Inhibition of Bax activity is crucial for the anti-apoptotic function of the human papillomavirus E6 oncoprotein. Oncogene, 25:4009-4015, 2006.

Yu J, Zhang L, Hwang PM, Kinzler KW, and Vogelstein B. PUMA induces the rapid apoptosis of colorectal cancer cells.. Molecular Cell, 7:673-682, 2001.

Zou H, Yang R, Hao J, Wang J, Sun C, and Fesik SW. Regulation of the Apaf-1/Caspase-9 apoptosome by Caspase-3 and XIAP. Journal of Biological Chemistry, 278:8091-8098, 2003.

Zha J, Lackner MR. Targeting the Insulin-like Growth Factor Receptor-1R Pathway for Cancer Therapy. Clin Cancer Res., 16(9):2512-7, 2010.
