# Supplementary material for: Efficient computation of minimal perturbation sets in gene regulatory networks
Source: Front Physiol. 2013 Dec 17;4:361. doi: 10.3389/fphys.2013.00361 (PMC3867968; doi:10.3389/fphys.2013.00361)
Supplement: Supplementary Table 2 — List of predicted miRNA with matching (and mis-matching) polarity, Cancer types, and supporting MIS IDs. [file DataSheet2.PDF]

Supplementary Table 2. List of predicted miRNA with matching (and mis-matching) polarity and Cancer types from literature (Sinha et al., 2008), and supporting MIS IDs (from Figure 4).

**Cancer**	**miRNA**	**Polarity**	**Reference**	**Match/Mismatch**	**MIS ID**
Breast cancer	hsa-miR-125b	Down	Iorio et al., 2005	Match	MIS 20
ColonTumor-derived cell line	hsa-miR-130a	Down	Gaur et al, 2007	Match	MIS 19
Prostate cancer	hsa-miR-141	Down	Porkka et al., 2007	Match	MIS 13
Colorectal neoplasia	hsa-miR-143	Down	Michael et al., 2003	Match	MIS 19
ColonTumor-derived cell line	hsa-miR-15a	Down	Gaur et al, 2007	Match	MIS 20
Chronic lymphocytic leukemia	hsa-miR-16	Down	Calin et al., 2004; Cimmino et al., 2005	Match	MIS 20
Glioblastoma	hsa-miR-181a	Down	Ciafre et al., 2005	Match	MIS 19
CNS Tumor-derived cell line	hsa-miR-181c	Down	Gaur et al, 2007	Match	MIS 19
Breast, colon, lung, pancreas, prostate, and stomach cancer	hsa-miR-200b	Down	Volinia et al., 2006	Match	MIS 20
Head and neck cancer cell lines	hsa-miR-200c	Down	Tran et al., 2007	Match	MIS 7
ColonTumor-derived cell line	hsa-miR-214	Down	Gaur et al, 2007	Match	MIS 19
Prostate cancer	hsa-miR-27a	Down	Porkka et al., 2007	Match	MIS 19
Hematologic Tumor-derived cell line	hsa-miR-27b	Down	Gaur et al, 2007	Match	MIS 19
Prostate cancer	hsa-miR-497	Down	Porkka et al., 2007	Match	MIS 20
CNS Tumor-derived cell line	hsa-miR-7	Down	Gaur et al, 2007	Match	MIS 19
Breast, colon, lung, pancreas, prostate, and stomach cancer	hsa-miR-95	Down	Volinia et al., 2006	Match	MIS 19
Head and neck cancer cell lines	hsa-let-7a	Up	Tran et al., 2007	Match	MIS 26
Breast, colon, lung, pancreas, prostate, and stomach cancer	hsa-let-7b	Up	Volinia et al., 2006	Match	MIS 26
Head and neck cancer cell lines	hsa-let-7c	Up	Tran et al., 2007	Match	MIS 26
Head and neck cancer cell lines	hsa-let-7d	Up	Tran et al., 2007	Match	MIS 26
Breast, colon, lung, pancreas,	hsa-let-7e	Up	Volinia et al., 2006	Match	MIS 24

prostate, and stomach cancer					
Head and neck cancer cell lines	hsa-let-7f	Up	Tran et al., 2007	Match	MIS 26
Head and neck cancer cell lines	hsa-miR-103	Up	Tran et al., 2007	Match	MIS 24
Colon, pancreas, prostate	hsa-miR-106a	Up	Volinia et al., 2006	Match	MIS 24
Cholangiocarcinoma cell line	hsa-miR-141	Up	Meng et al., 2006	Match	MIS 24
Head and neck cancer cell lines	hsa-miR-15a	Up	Tran et al., 2007	Match	MIS 24
Head and neck cancer cell lines	hsa-miR-15b	Up	Tran et al., 2007	Match	MIS 24
Head and neck cancer cell lines	hsa-miR-16	Up	Tran et al., 2007	Match	MIS 24
Prostate cancer	hsa-miR-202	Up	Porkka et al., 2007	Match	MIS 24
Head and neck cancer cell lines	hsa-miR-22	Up	Tran et al., 2007	Match	MIS 22
B-cell lymphoma	hsa-miR-221	Up	Esquela-Kerscher and Slack, 2006	Match	MIS 24
Head and neck cancer cell lines	hsa-miR-27a	Up	Tran et al., 2007	Match	MIS 22
Head and neck cancer cell lines	hsa-miR-30b	Up	Tran et al., 2007	Match	MIS 24
Colon, pancreas, prostate	hsa-miR-30c	Up	Volinia et al., 2006	Match	MIS 24
Breast, colon, lung, pancreas, prostate, and stomach cancer	hsa-miR-30d	Up	Volinia et al., 2006	Match	MIS 24
Head and neck cancer cell lines	hsa-miR-320	Up	Tran et al., 2007	Match	MIS 24
Prostate cancer	hsa-miR-498	Up	Porkka et al., 2007	Match	MIS 22
Head and neck cancer cell lines	hsa-miR-98	Up	Tran et al., 2007	Match	MIS 24
Lung cancer	hsa-miR-17	Down	Takamizawa et al., 2004; Johnson et al., 2005;Hayashita et al., 2005; O'Donnell et al., 2005	Mismatch	MIS 19
Head and neck cancer cell lines	hsa-miR-24	Down	Tran et al., 2007	Mismatch	MIS 19
Colorectal neoplasia	hsa-miR-31	Down	Bandres et al., 2006	Mismatch	MIS 19
Colorectal neoplasia	hsa-miR-96	Down	Bandres et al., 2006	Mismatch	MIS 19
Breast, colon, lung, pancreas, prostate, and stomach cancer	hsa-let-7g	Up	Volinia et al., 2006	Mismatch	MIS 26
CNS Tumor-derived cell line	hsa-let-7i	Up	Gaur et al, 2007	Mismatch	MIS 26

CNS Tumor-derived cell line	hsa-miR-149	Up	Gaur et al, 2007	Mismatch	MIS 24
CNS Tumor-derived cell line	hsa-miR-181b	Up	Gaur et al, 2007	Mismatch	MIS 4
Prostate cancer	hsa-miR-19b	Up	Porkka et al., 2007	Mismatch	MIS 22
Prostate cancer	hsa-miR-26a	Up	Porkka et al., 2007	Mismatch	MIS 4
CNS Tumor-derived cell line	hsa-miR-324-3p	Up	Gaur et al, 2007	Mismatch	MIS 24
Breast, colon, lung, pancreas, prostate, and stomach cancer	hsa-miR-34a	Up	Volinia et al., 2006	Mismatch	MIS 24
Head and neck cancer cell lines	hsa-miR-375	Up	Tran et al., 2007	Mismatch	MIS 2
ColonTumor-derived cell line	hsa-miR-422a	Up	Gaur et al, 2007	Mismatch	MIS 22
ColonTumor-derived cell line	hsa-miR-424	Up	Gaur et al, 2007	Mismatch	MIS 24

References

Bandres E, Cubedo E, Agirre X, Malumbres R, Zarate R, Ramirez N, Abajo A, Navarro A, Moreno I, Monzo M et al : Identification by Real-time PCR of 13 mature microRNAs differentially expressed in colorectal cancer and non-tumoral tissues. Mol Cancer 2006, 5:29.

Calin GA, Liu CG, Sevignani C, Ferracin M, Felli N, Dumitru CD, Shimizu M, Cimmino A, Zupo S, Dono M et al : MicroRNA profiling reveals distinct signatures in B cell chronic lymphocytic leukemias. Proc Natl Acad Sci U S A 2004, 101(32):11755-11760.

Ciafre SA, Galardi S, Mangiola A, Ferracin M, Liu CG, Sabatino G, Negrini M, Maira G, Croce CM, Farace MG: Extensive modulation of a set of microRNAs in primary glioblastoma. Biochem Biophys Res Commun 2005, 334(4):1351-1358.

Cimmino A, Calin GA, Fabbri M, Iorio MV, Ferracin M, Shimizu M, Wojcik SE, Aqeilan RI, Zupo S, Dono M et al : miR-15 and miR-16 induce apoptosis by targeting BCL2. Proc Natl Acad Sci U S A 2005, 102(39):13944-13949.

Esquela-Kerscher A, Slack FJ: Oncomirs - microRNAs with a role in cancer. Nat Rev Cancer 2006, 6(4):259-269

Gaur A, Jewell DA, Liang Y, Ridzon D, Moore JH, Chen C, Ambros VR, Israel MA: Characterization of microRNA expression levels and their biological correlates in human cancer cell lines. Cancer Res 2007, 67(6):2456-2468.

Hayashita Y, Osada H, Tatematsu Y, Yamada H, Yanagisawa K, Tomida S, Yatabe Y, Kawahara K, Sekido Y, Takahashi T: A polycistronic microRNA cluster, miR-17-92, is overexpressed in human lung cancers and enhances cell proliferation. Cancer Res 2005, 65(21):9628-9632.

Iorio MV, Ferracin M, Liu CG, Veronese A, Spizzo R, Sabbioni S, Magri E, Pedriali M, Fabbri M, Campiglio M et al : MicroRNA gene expression deregulation in human breast cancer. Cancer Res 2005, 65(16):7065-7070.

Johnson SM, Grosshans H, Shingara J, Byrom M, Jarvis R, Cheng A, Labourier E, Reinert KL, Brown D, Slack FJ: RAS is regulated by the let-7 microRNA family. Cell 2005, 120(5):635-647.

Meng F, Henson R, Lang M, Wehbe H, Maheshwari S, Mendell JT, Jiang J, Schmittgen TD, Patel T: Involvement of human micro-RNA in growth and response to chemotherapy in human cholangiocarcinoma cell lines. Gastroenterology 2006, 130(7):2113-2129.

Michael MZ, SM OC, van Holst Pellekaan NG, Young GP, James RJ: Reduced accumulation of specific microRNAs in colorectal neoplasia. Mol Cancer Res 2003, 1(12):882-891.

O'Donnell KA, Wentzel EA, Zeller KI, Dang CV, Mendell JT: c-Myc-regulated microRNAs modulate E2F1 expression. Nature 2005, 435(7043):839-843.

Porkka KP, Pfeiffer MJ, Waltering KK, Vessella RL, Tammela TL, Visakorpi T: MicroRNA expression profiling in prostate cancer. Cancer Res 2007, 67(13):6130-6135.

Sinha, A. U., Kaimal, V., Chen, J., and Jegga, A. G.: Dissecting microregulation of a master regulatory network. BMC Genomics 2008, 9.

Takamizawa J, Konishi H, Yanagisawa K, Tomida S, Osada H, Endoh H, Harano T, Yatabe Y, Nagino M, Nimura Y et al : Reduced expression of the let-7 microRNAs in human lung cancers in association with shortened postoperative survival. Cancer Res 2004, 64(11):3753-3756.

Tran N, McLean T, Zhang X, Zhao CJ, Thomson JM, O'Brien C, Rose B: MicroRNA expression profiles in head and neck cancer cell lines. Biochem Biophys Res Commun 2007, 358(1):12-17.

Volinia S, Calin GA, Liu CG, Ambs S, Cimmino A, Petrocca F, Visone R, Iorio M, Roldo C, Ferracin M et al : A microRNA expression signature of human solid tumors defines cancer gene targets. Proc Natl Acad Sci U S A 2006, 103(7):2257-2261.
