# Supplementary material for: Efficient computation of minimal perturbation sets in gene regulatory networks
Source: Front Physiol. 2013 Dec 17;4:361. doi: 10.3389/fphys.2013.00361 (PMC3867968; doi:10.3389/fphys.2013.00361)
Supplement: Supplementary Table 3 — Simulation results of computing MIS patterns on Random Networks of varying complexities. [file DataSheet3.PDF]

Supplementary Table 3. Simulation results of our algorithms for computing MIS patterns on Random Networks of different sizes. The results are reported for 10 Random Networks of each size. MIS patterns were generated for the same desired steady state for all the networks of a given size. Simulations were performed on a 2.7 GHz Mac OS desktop with a cut-off time limit of 2h. Not all the simulations could finish computing the MIS patterns, and reported only partial sets of MIS patterns within the cut-off limit. There were 6/10 networks of size 100 nodes, 3/10 of size 80 nodes and 1/10 of size 60 nodes finished generating the MIS patterns from the unrolled network but were still simulating the generated patterns to test for minimality at the cut-off limit. All the networks of size 20 and 40 nodes finished simulations well within the cut-off time. The average computation time of simulations, reported below, include the 2h cut-off time of the networks that could not finish simulating all the MIS patterns. Number of MIS patterns for networks of a given size can vary significantly. The column 4 in the table report the maximum and minimum number of MIS patterns reported over the 10 different networks of a given size. Random Networks used here are available along with the software binaries online.

**Nodes**	**Edges (Avg)**	**Avg. Time (in sec)**	**Number of MIS Maximum (Minimum)**
20	38.6	80	75 (9)
40	75.9	546	145 (2)
60	109.3	1064	105 (1)
80	141.2	1845	383 (0)
100	189.1	4988	330 (20)
